# Comparison of the Nutritional Value of Locally Formulated Food Preparations and Standard Ready‐to‐Use Therapeutic Foods for the Management of Uncomplicated Severe Acute Malnutrition in Children Under Five in the Democratic Republic of the Congo

**DOI:** 10.1002/fsn3.71302

**Published:** 2025-12-08

**Authors:** Marc Bosonkie, Celestin Nzanzu Mudogo, Hannah Silverstein, Olufunmilayo I. Fawole, Gael Compta, Koto‐Te‐Nyiwa Ngbolua, Tesky Koba, Ruphin Mbuyi, Berthold Bondo, Paul‐Samsom Lusamba, Mala Ali Mapatano

**Affiliations:** ^1^ Department of Nutrition, Kinshasa School of Public Health, School of Medicine University of Kinshasa Kinshasa Democratic Republic of the Congo; ^2^ Faculty of Medicine, Department of Basic Sciences University of Kinshasa Kinshasa Democratic Republic of the Congo; ^3^ School of Social Work University of North Carolina at Chapel Hill Chapel Hill North Carolina USA; ^4^ Faculty of Public Health, Department of Epidemiology and Medical Statistics, College of Medicine University of Ibadan Ibadan Nigeria; ^5^ Department of International Health and Sustainable Development Celia Scott Weatherhead School of Public Health at Tulane University New Orleans Louisiana USA; ^6^ Faculty of Science, Department of Biology University of Kinshasa Kinshasa Democratic Republic of the Congo; ^7^ Research and Training Management in Public Health, National Institute of Public Health Kinshasa Democratic Republic of the Congo; ^8^ Centre National d'Appui au Développement et à la Participation Populaire, Civil Society Organizations Kinshasa Democratic Republic of the Congo; ^9^ Barumbu General Hospital Kinshasa Democratic Republic of the Congo; ^10^ Department of Biostatistics and Epidemiology, Kinshasa School of Public Health, School of Medicine University of Kinshasa Kinshasa Democratic Republic of the Congo

**Keywords:** Codex Alimentarius, COVID‐19, Democratic Republic of the Congo, locally formulated food preparations, ready‐to‐use therapeutic foods, uncomplicated severe acute malnutrition

## Abstract

Severe acute malnutrition (SAM) remains a major concern in sub‐Saharan Africa. In the Democratic Republic of the Congo (DRC), health care providers in therapeutic feeding units produce locally formulated food preparations empirically, with unknown macro‐ and micronutrient compositions. This study compares ingredients and nutritional values of locally formulated food preparation with those of standard ready‐to‐use therapeutic foods, as per Codex Alimentarius standards. A descriptive, comparative case study design was employed across outpatient therapeutic nutrition units (OTNUs) in four provinces of the DRC. Food samples were collected. Analysis included theoretical nutritional values calculation using NutVal software and laboratory analyses for selected nutrients (phosphorus, iron, calcium, magnesium, energy, lipids, and proteins) via standard protocols. From theoretical nutritional values, we calculate percentages of WHO/Codex Alimentarius requirements covered by the recipes and recipes in deficit by nutrient group. Laboratory analyses of selected nutrients were compared with the cut‐off of the corresponding nutrient in the Codex Alimentarius. Analyses were conducted using Stata 18. Locally formulated food preparations were mostly milk‐ and peanut‐free, often exceeding protein and lipid recommendations but deficient in vitamins (A, D, E, K, B12, folate), and critical minerals (calcium, iron, iodine, selenium, zinc). Compliance with Codex standards was very low, confirming macronutrient inadequacy but widespread micronutrient gaps. This study highlights critical gaps in locally formulated preparations in the management of SAM compared with WHO/Codex standards, emphasizing the need for fortification, balanced nutrients, and regulatory oversight. Engaging local industries, optimizing ingredient value chains, and rigorous evaluation are essential to ensure sustainable, effective, and context‐appropriate SAM treatment solutions.

## Graphical abstract

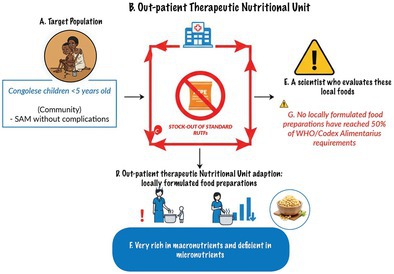

## 1 Introduction

Malnutrition remains one of the major public health challenges in many low‐ and middle‐income countries (UNICEF 2023). Approximately 45 million children under the age of five, accounting for approximately 6.8% of this age group, are severely malnourished worldwide (UNICEF, WHO, and World Bank 2023). Severe acute malnutrition (SAM) remains a significant concern in sub‐Saharan Africa (Daures 2024; Rohloff et al. 2024; World Health Organization 2024). In West and Central Africa alone, approximately 16.7 million children under five suffer from acute malnutrition, with one‐third experiencing its most severe forms (Food and Agriculture Organization, United Nations Economic Commission for Africa, and African Union Commission 2021). SAM is associated with a high risk of mortality in children under 5 years of age (McDonald et al. 2013). In addition to its disadvantageous effects on children's psychomotor development, SAM is a long‐term risk to the education and economic performance of affected children (Mwene‐Batu et al. 2020).

To treat SAM, both locally made food preparations and standard ready‐to‐use therapeutic foods (RUTFs) aim to provide safe, high‐nutrient, and culturally accepted diets that support catch‐up growth and recovery. Despite this shared objective, there are notable differences. Industrial RUTFs are produced to rigorous, uniform standards set by WHO/UNICEF, which ensure quality, safety, and a long shelf life (Briend et al. 2015). Conversely, locally prepared foods are produced from regionally available ingredients, resulting in variations in nutritional makeup, safety, and storage stability (Briend et al. 2015). In this context, nurses prepare these in health centre kitchens, sometimes including cow's milk, peanuts, animal protein. These local alternatives are usually more affordable and well‐accepted within communities and can foster local empowerment and sustainability, though they often lack robust quality controls and consistency (Pajak et al. 2025).

According to Codex Alimentarius and recent WHO guidelines (FAO/WHO 2022; WHO 2023), standard RUTFs are defined as energy‐dense pastes delivering 520–550 kcal per 100 g, with moisture content below 2.5%. Protein should provide 10%–12% of total energy (2.5–3.0 g/100 kcal), with at least half from milk, and a PDCAAS of ≥ 0.9. Fats should account for 45%–60% of total energy (5.0–7.0 g/100 kcal). Essential vitamins and minerals must be present in bioavailable forms—such as calcium (55–151 mg/100 kcal), magnesium (15–45 mg/100 kcal), iron (1.8–2.7 mg/100 kcal), among others (WHO 2023). The World Health Assembly's 2025 targets are to reduce the prevalence of wasting to less than 5%. In the Democratic Republic of the Congo (DRC), the prevalence of wasting decreased only slightly between 2023 and 2024 from 8% to 7% overall, and from 3% to 2% for SAM (EDS 2024; Mwene‐Batu et al. 2020). This slow progress indicates that the country remains off track.

The DRC addresses SAM through its national Integrated Management of Acute Malnutrition (IMAM) protocol, revised in 2022. Coordination falls to PRONANUT within the Ministry of Public Health, covering policy, resource mobilization, supply logistics, and data management. Provincial health divisions adapt these strategies into actionable guidelines and supervise implementation at the district level, where health management teams and district medical officers oversee care delivery. At the service level, outpatient therapeutic feeding units (OTFUs) handle uncomplicated SAM, while referral hospitals manage more severe cases. Community‐level staff, including nurses and community health workers, are responsible for screening, referrals, follow‐up, and household education (MSPHP 2022).

The COVID‐19 pandemic starkly revealed the vulnerability of the DRC's food supply chains, resulting in critical shortages of imported RUTFs, vaccines, and essential drugs (Bosonkie et al. 2025). Locally produced foods became key alternatives, though their nutritional adequacy is not well documented. Such preparations bring benefits like promoting biodiversity, improving affordability, and enhancing cultural suitability, but they also present challenges regarding Codex compliance, antinutritional factors, and certification (Pajak et al. 2025). In addition, conflict, displacement, and adverse weather events in regions like North Kivu disrupt markets, damage farmland, and limit the availability of key dietary components (UNOCHA 2024). Lengthy supply routes and high transportation costs push food prices higher, while poverty curtails purchasing power (World Bank 2023). Consequently, a nutritious diet often remains inaccessible, and health services for nutrition support are limited in remote areas (WFP 2024).

Despite being pivotal in SAM care, little is known about the nutritional adequacy of the DRC's locally made food preparations. Often developed by trial and error with no formal nutrient analysis, these foods may be deficient or imbalanced in macro‐ and micronutrients, risking ineffective treatment, deficiencies, metabolic issues, developmental setbacks, or increased mortality (Black et al. 2013). Meeting international standards is thus essential for safe and effective nutritional recovery (FAO/WHO 2022). This study aims to compare the ingredients and nutritional profiles of locally formulated foods used in four provinces with Codex Alimentarius standards, utilizing both NutVal‐based calculations and lab analyses. Given the highest COVID‐19 burdens occurred in Haut‐Katanga, Kinshasa, Kongo‐Central, and North Kivu during the first four waves (March 2020–December 2022), the study focuses on these provinces in the DRC (Kashiya et al. 2023; Otshudiema et al. 2022).

## 2 Materials and Methods

### 2.1 Study Site and Design

This study was conducted in outpatient therapeutic nutrition units (OTNUs) in four provinces of the Democratic Republic of the Congo, namely, Haut‐Katanga, Kinshasa, Kongo‐Central, and North Kivu, in November 2022. This study employed a descriptive, comparative case study design to compare the ingredients and the nutritional values of these formulations against established international standards for SAM as outlined by the Codex Alimentarius.

### 2.2 Sampling

The study surveyed OTNUs operating through the four COVID‐19 waves. A total of 104 units preparing local foods were identified: 24 in Kinshasa, 27 in Kongo‐Central, 23 in Haut‐Katanga, and 30 in North Kivu. The sampling frame consisted of all OTNUs listed in alphabetical order. Simple random sampling selected 40 OTNUs from both urban and rural districts: 10 in Haut‐Katanga, 8 in Kinshasa, 10 in Kongo‐Central, and 12 in North Kivu.

### 2.3 Data Collection

#### 2.3.1 Recruitment and Training of Research Assistants

We recruited four research assistants from the Faculty of Science, Department of Biology, who had been working on food science and nutrition for at least 3 years. The research assistants were trained for two days by the research team on the following topics: the problem statement, methodology, the ODK (Open Data Kit) application for data recording, and sample collection, packaging, and transportation.

#### 2.3.2 Pretest

Before the start of the survey, a pretest of the study process on six participants was conducted for a day in two OTNUs in Kinshasa. The pretest helped ensure the quality of the food samples collected, their packaging, and their mode of transport.

### 2.4 Sample Collection Techniques

On the field, food preparations were made by nutritionists (Bachelor's degree, 3 years) responsible for the OTNU. The same preparations were then replicated in the laboratory, following the same procedures, by laboratory assistants (Master's degree in Biology, 7 years of study) before analysis using raw materials. Research assistants recorded each cooked food, specifying the quantity and cost, cooking method and time, and amount consumed per serving. The foods were prepared in the presence of the data collector to allow for the recording of the procedure and the total cooking time for replication in the laboratory in Kinshasa. The samples were packaged in food‐grade polypropylene (PP) plastic bowls with a capacity of 250 mL. PP was chosen because it is inert for food storage and does not release compounds likely to alter the nutritional composition. The insulated coolers used were equipped with ice packs, which were replaced every 12 h. An electronic thermometer (Testo, Germany) was used to monitor the internal temperature, which was maintained between +2 and +8°C throughout transportation. Standardization of portions was ensured by using three spoonfuls which correspond to a measure validated by the National Nutrition Program (PRONANUT, DRC), averaging 30 g of preparation per serving. This was chosen to ensure homogeneity of sampling and a reliable comparison between recipes.

Since the laboratory was located in Kinshasa, samples from remote sites (Haut‐Katanga and North Kivu) were transported by air, with a maximum duration of 48 h between collection and arrival at the laboratory. During transport, the samples were kept in sealed insulated coolers, and temperature was monitored using electronic probes. For Kongo‐Central, located approximately 300 km from Kinshasa, samples were transported by road using a dedicated vehicle.

For each analysis, three measurements were performed on the same sample, and the average value was calculated for protein, lipid, and spectrometry analyses. Given resource constraints and the need to align with international standards, we limited our analysis to energy, protein, fat, phosphorus, magnesium, iron, and calcium. The profiles of these seven nutrients for each recipe are presented in Table S1. A full nutrient profile analysis (covering all vitamins and minerals) requires extensive laboratory resources and funding, which were beyond the scope of this study. These parameters were selected because they are the primary determinants of therapeutic adequacy in SAM management, directly influencing weight gain, tissue repair, and reduction of mortality risk. For the full nutrients profile (energy, protein, fat, calcium, copper, iodine, iron, magnesium, selenium, zinc, vitamin A, thiamine, riboflavin, niacin, pantothenic acid, pyridoxine, folate, vitamin B12, vitamin C, vitamin D, vitamin E, and vitamin K), their values were estimated theoretically using the NutVal software. The profiles of these twenty‐two nutrients for each recipe are presented in Table S2.

Portion sizes for SAM therapy were not standardized, as each OTNU followed its own rules. Typically, mothers received a two‐week ration of 1 kg maize, 0.5 kg soybean flour, 400 g milk, 450 g margarine, and 300 g sugar, among other items (details in Table 1). After two weeks, mothers would return with their children for reassessment and to collect a new package. For a 14‐day package, the cost was approximately 2300 Congolese francs (≈US$10).

**TABLE 1:** Minimum and maximum values of nutrients in the nutritional composition of RUTFs defined by the Codex Alimentarius.

Nutrient	Unit	Minimum	Maximum
Energy	kcal/100 g	520	550
Protein	g/100 kcal	2.5	3.0
Fat	g/100 kcal	5	7
Calcium	mg/100 kcal	55	151
Copper	mg/100 kcal	0.25	0.35
Iodine	μg/100 kcal	13	27
Iron	mg/100 kcal	1.8	2.7
Magnesium	mg/100 kcal	15	45
Selenium	μg/100 kcal	3.6	8
Zinc	mg/100 kcal	2	2.7
Vitamin A	μg RE/100 kcal	145	308
Thiamine	mg/100 kcal	0.09	—
Riboflavin	mg/100 kcal	0.29	—
Niacin	mg/100 kcal	0.91	—
Pantothenic acid	mg/100 kcal	0.55	—
Pyridoxin	mg/100 kcal	0.11	—
Folate	μg/100 kcal	36	—
Vitamin B12	μg/100 kcal	0.29	—
Vitamin C	mg/100 kcal	9	—
Vitamin D	μg/100 kcal	2.7	4.2
Vitamin E	mg α‐TE/100 kcal	3.6	—
Vitamin K	μg/100 kcal	2.7	6

### 2.5 Supervision of Field Teams

To ensure the integrity of the data collected, rigorous supervision was implemented. The four technical staff involved in the study maintained constant communication with the teams throughout the procurement process, as well as during the sampling phase.

### 2.6 Determination of Nutrient Composition

The nutrient composition of the collected food samples was analyzed in accordance with established standard protocols.

#### 2.6.1 Determination of Macronutrient Content

Total protein determination was conducted via the KJELDAHL method (Ummah 2019). This method involves the conversion of organic nitrogen into ammonia through the application of oxidants and catalysts, followed by a chemical and titration process to evaluate NH4+ concentrations. The total lipid content was assessed via the Soxhlet method described by Saini et al., which facilitates the extraction of lipids by employing suitable organic solvents (Saini et al. 2021). Energy value calculations were performed by determining the caloric energy of 100 g of samples via ATWATER coefficients for proteins, lipids, and carbohydrates, as indicated by Trèche et al. (1995) and (Sánchez‐Peña et al. 2017).

#### 2.6.2 Determination of Mineral Content

Mineral (phosphorus, magnesium, iron, and calcium) detection and quantification were executed via X‐ray fluorescence spectroscopy. For this analysis, powdered food samples (five grams) were compressed into pellets via hydraulic presses and examined with fluorescent spectrometers (Pozza et al. 2024).

### 2.7 Study Variables

For laboratory perspectives: the assessment focused on four micronutrients—phosphorus, iron, calcium, and magnesium—and three macronutrients, energy, lipids, and proteins. By using NutVal, they were considered as variables: energy, protein, fat, calcium, copper, iodine, iron, magnesium, selenium, zinc, vitamin A, thiamine, riboflavin, niacin, pantothenic acid, pyridoxine, folate, vitamin B12, vitamin C, vitamin D, vitamin E, and vitamin K. The acceptable ranges defined by the Codex are displayed in Table 1, which summarizes the minimum and maximum values of nutrients in the nutritional composition of RUTFs.

Some definitions are as follows:

- Fat‐soluble vitamins are a group of vitamins (A, D, E, and K) that dissolve in fats and oils and are stored in the liver and fatty tissues of the body (WHO and FAO 2024).
- Critical minerals are essential inorganic elements required in small amounts for human health, growth, and development. They play key roles in enzyme function, oxygen transport, bone health, immune response, and cellular signaling. In nutrition, the most frequently emphasized “critical minerals” include iron, zinc, calcium, iodine, selenium, and magnesium deficiencies of which are strongly linked to growth faltering, anemia, impaired cognitive development, and higher child morbidity and mortality (WHO and FAO 2024).
- The Codex Alimentarius is a collection of internationally recognized standards, guidelines, and codes of practice related to food safety and quality. It was established by the Codex Alimentarius Commission, which is jointly run by the World Health Organization (WHO) and the Food and Agriculture Organization (FAO) of the United Nations.

### 2.8 Statistical Analysis

Forty samples of locally formulated food preparations were analyzed to evaluate their micronutrient and macronutrient profiles compared with the standards established by the Codex Alimentarius, as outlined by the Food and Agriculture Organization (FAO) and WHO.

**FIGURE 1:**
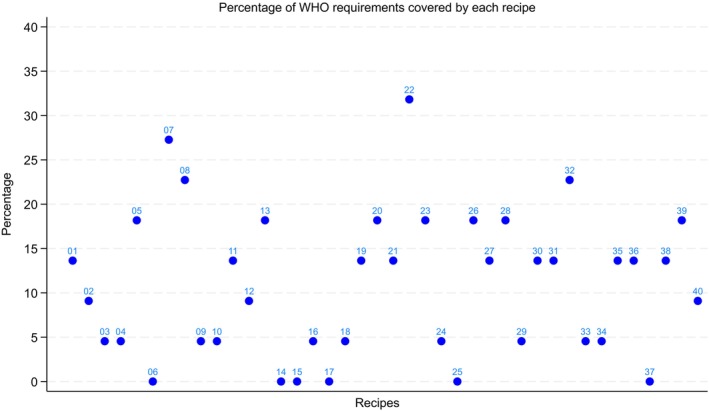
Percentage of WHO/Codex Alimentarius requirements covered by each recipe.

**FIGURE 2:**
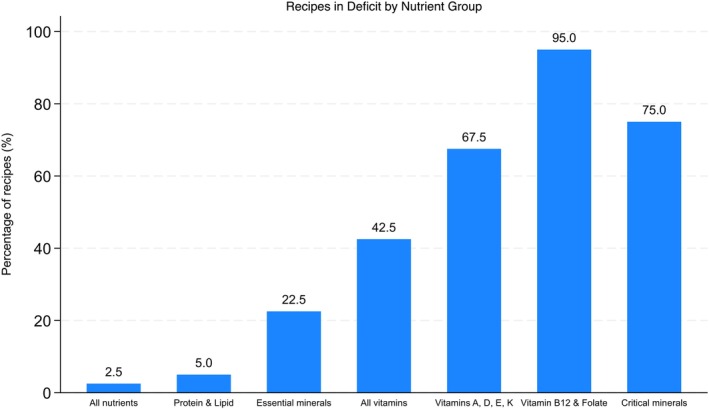
Recipes in deficit by nutrient group.

Using the NutVal software, we estimated the theoretical nutritional value of each recipe by imputing the ingredients and corresponding daily rations (Kcal/person/day) collected for each recipe. NutVal allowed us to automatically calculate nutritional values based on those ingredients, including energy density, proteins, and lipids, as well as a range of vitamins and minerals. Based on these results, we created figures comparing the nutritional content of each recipe with the WHO guidelines for RUTF (WHO 2023) and Codex Alimentarius (FAO and WHO 2022). We then developed a summary variable showing the number of components for which each recipe was within the thresholds and ranked the recipes accordingly. Using the nutritional values of the recipes and the WHO thresholds for RUTF, we captured the number of nutrients falling below or above the recommended ranges for each recipe as in Figure 1. We then made a summary figure that grouped recipes according to specific patterns of nutrient deficits, including deficiencies across all essential nutrients, deficits limited to protein and lipids, essential minerals, vitamins, fat‐soluble vitamins, vitamin B12 and folate, and critical minerals as in Figure 2.

Based on laboratory analyses (measured values), we developed visual representations illustrating the levels of both micronutrients and macronutrients of selected nutrients across all the samples. Each of the seven figures delineates the Codex‐defined minimum and maximum values, with samples that fall within the acceptable range highlighted in green. The samples that exceed the maximum threshold are denoted in orange, whereas those that fall below the minimum threshold are indicated in red. Furthermore, the mean value for each nutrient is represented as a triangle within the figures.

Additionally, we presented the mean values and confidence intervals for each component across the 40 samples. We conducted two‐tailed *t*‐tests to assess whether the mean values conformed to the acceptable minimum and maximum standards outlined by the Codex Alimentarius (FAO and WHO 2022). All analyses were performed via Stata 18 software.

### 2.9 Ethical Considerations

Before initiating data collection, we obtained ethical approval from the Ethics Committee of the Kinshasa School of Public Health (Ref: *ESP/CE/089/2022*). We maintained the strict confidentiality and anonymity of all participants. Informed consent was obtained from each OTFU official before participation. The respondents were fully informed of their right to withdraw from the interview at any time without any consequences. The interviews were conducted in the language with which the participants were most comfortable, ensuring clear communication.

Additionally, participants had the right to withhold information regarding the names of local foods used in their OTNUs as part of their resilience strategies. Identifying details such as the OTNU's name, manager's phone number, and address were collected; these data were securely encrypted and stored on a password‐protected server to ensure confidentiality and data protection.

## 3 Results

Our results are presented in the following order: (1) the ingredients used in each locally formulated food preparation; (2) macronutrient and micronutrient profiles according to NutVal (theoretical values); and (3) selected macronutrient and micronutrient profiles based on laboratory analyses (measured values).

More than three‐quarters of OTNUs produced locally formulated food preparations without using cow's milk, more than two‐thirds without peanuts, and nearly one‐fifth with an animal protein source (Table 2).

**TABLE 2:** Locally formulated food preparations per province and ingredients used.

Code	Locally formulated food preparations	Province	Use of cow's milk	Use of peanuts	Use of animal protein
MES 01	Wheat + Soybean + Bicarbonate + Margarine + Milk + Sugar	Kinshasa	Yes	No	No
MES 02	Wheat + White Maize + Yellow Maize + Soybean + Millet + Margarine + Milk + Sugar	Kinshasa	Yes	No	No
MES 03	Maize + Caterpillar + Rice + Soybean + Oil + Salt + Sugar + Milk	Kinshasa	Yes	No	Yes
MES 04	Wheat + Caterpillar + Rice + Soybean + Oil + Salt + Sugar + Milk	Kinshasa	Yes	No	Yes
MES 05	Maize + Soybean	Kinshasa	No	No	No
MES 06	Peanut + Sugar + Oil	Kinshasa	No	Yes	No
MES 07	Maize + Soybean + Avocado + Milk + Sugar	Kinshasa	Yes	No	No
MES 08	Maize + Soybean + Avocado + Egg + Sugar	Kinshasa	No	No	No
MES 09	Maize + Soybean + Sorghum	Nord Kivu	No	No	No
MES 10	Maize + Wheat + Sorghum + Oil + Sugar	Nord Kivu	No	No	No
MES 11	Maize + Sorghum + Soybean + Millet	Nord Kivu	No	No	No
MES 12	Maize + Peanut Paste + Sugar	Nord Kivu	No	Yes	No
MES 13	Maize + Soybean + Sugar	Nord Kivu	No	No	No
MES 14	Milk + Oil + Sugar	Nord Kivu	Yes	No	No
MES 15	Sweet Potato + Peanut Paste + Spices + Palm Oil	Nord Kivu	No	Yes	No
MES 16	Vegetables + Oil + Peanut Paste + Spices + Small Fish + (Maize + Cassava)	Nord Kivu	No	Yes	Yes
MES 17	Potato + Soybean + Spices + Palm Oil	Nord Kivu	No	No	No
MES 18	Peanut Paste	Nord Kivu	No	Yes	No
MES 19	Soy Milk + Egg	Nord Kivu	Yes	No	No
MES 20	Cassava Leaves + Peanut + Small Fish + Fufu (Maize)	Nord Kivu	No	Yes	Yes
MES 21	Peanut + Maize	Haut Katanga	No	Yes	No
MES 22	Maize + Soybean + Sugar	Haut Katanga	No	No	No
MES 23	Maize + Soybean + Sugar	Haut Katanga	No	No	No
MES 24	Rice + Peanut	Haut Katanga	No	Yes	No
MES 25	Soybean + Maize + Oil + Sugar	Haut Katanga	No	No	No
MES 26	Soybean + Rice + Sugar	Haut Katanga	No	No	No
MES 27	Soybean + Milk + Maize + Peanut	Haut Katanga	No	Yes	No
MES 28	Maize + Soybean	Haut Katanga	No	No	No
MES 29	Maize + Soybean + Small Fish + Salt + Oil	Haut Katanga	No	No	Yes
MES 30	Green Peas + Maize	Haut Katanga	No	No	No
MES 31	Maize + Soybean	Kongo Central	No	No	No
MES 32	Maize + Soybean + Caterpillar	Kongo Central	No	No	Yes
MES 33	Maize + Soybean + Caterpillar	Kongo Central	No	No	Yes
MES 34	Maize + Soybean	Kongo Central	No	No	No
MES 35	Wheat + Soybean	Kongo Central	No	No	No
MES 36	Maize + Peanut	Kongo Central	No	Yes	No
MES 37	Wheat + Peanut Paste + Sugar + Milk + Oil	Kongo Central	Yes	Yes	No
MES 38	Wheat + Soybean + Blue Band (Margarine) + Milk + Sugar	Kongo Central	Yes	No	No
MES 39	Maize + Soybean	Kongo Central	No	No	No
MES 40	Cassava + Peanut Paste	Kongo Central	No	Yes	No

According to NutVal (theoretical values), out of 40 locally formulated food preparations, the median compliance was 3 nutrients out of 22 (13.64%). No locally formulated food preparation reached ≥ 50% compliant nutrients (≥ 11/22). Only one formulation (MES 22) reaches 7/22 (31.82%), and another (MES 07) reaches 6/22 (27.27%). Three out of four recipes contained excess protein, while one out of three displayed excess lipid levels, highlighting a tendency toward macronutrient oversupply. Refer to Figure 1 below for further illustration.

In contrast, the major gaps are micronutritional. Almost all recipes were deficient in vitamin B12 and folate (95%). Deficits were also frequent for the fat‐soluble vitamins A, D, E, and K (67.5%). Furthermore, three quarters of recipes showed shortfalls in critical minerals. Refer to Figure 2 below for further illustration. Based on laboratory analyses (measured values), acknowledging that certain nutrients were not analyzed owing to financial limitations, with the analyzed nutrients, the tendency of our locally formulated food preparations to be rich in macronutrients and deficient in micronutrients is consistent with theoretical values. More than 90% of the samples (orange circles) exceed the upper limit of the recommended protein range, indicating that most recipes contained excessive protein levels.

**FIGURE 3:**
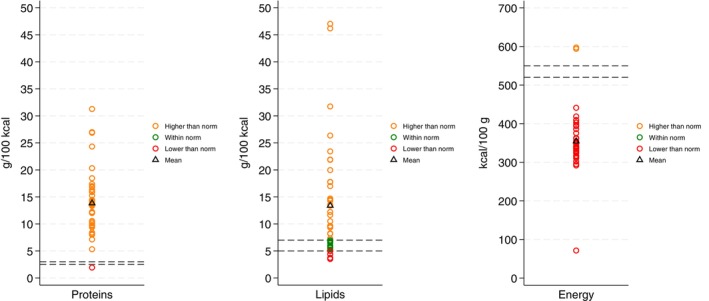
Macronutrient content of locally formulated food preparations (proteins, lipids, and energy).

Lipid profile analysis revealed that fewer than 10% of the samples (indicated by red circles) presented lipid levels below the established standard. Approximately 20% of the samples (represented by green circles) fell within the recommended range, demonstrating adherence to nutritional guidelines. Conversely, approximately 70% of the samples (denoted by orange circles) surpassed the upper limit, revealing an excessively high lipid content in the majority of the samples. Over 90% of the samples, represented by red circles, fell below the recommended standard's lower threshold in terms of energy content. This finding underscores a pervasive deficiency in energy density (Figure 3).

**FIGURE 4:**
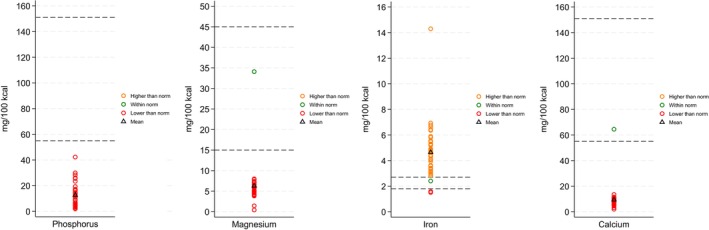
Micronutrient content of locally formulated food preparations (phosphorus, magnesium, iron, and calcium).

In the analysis of phosphorus, magnesium, and calcium levels, the majority of values were significantly below the minimum threshold, as indicated by the red markers. This finding suggests that locally formulated food preparations generally exhibit deficiencies in these essential minerals. Conversely, more than 90% of the samples, represented by orange circles, surpassed the upper limit of the recommended range for iron. This finding indicates that excess iron is prevalent in locally formulated food preparations (Figure 4).

The analysis of locally formulated food preparations revealed significant deviations from the Codex Alimentarius standards across both micronutrients and macronutrients. The phosphorus (*t* = −30.04, *p* < 0.05), magnesium (*t* = −11.56, *p* < 0.05), and calcium (*t* = −31.08, *p* < 0.05) levels were all significantly lower than the recommended ranges of 55–151 and 15–45 mg/100 g, respectively. In contrast, iron levels were significantly higher than the Codex range (1.8–2.7 mg/100 g) (*t* = 8.73, *p* < 0.05).

With respect to macronutrients, the lipid (*t* = 5.14, *p* < 0.05) and protein (*t* = 11.99, *p* < 0.05) contents exceeded the recommended ranges of 5–7 and 2.5–3.0 g/100 g, respectively. Moreover, the energy content of locally formulated food preparations was significantly lower than the Codex standard (520–550 kcal/100 g) (*t* = −13.20, *p* < 0.05) (Tables 2 and 3).

**TABLE 3:** Comparison of locally produced RUTFs and Codex Alimentarius standards.

	Mean	Confidence interval	Codex Alimentarius RUTF		*t* test	
			Minimum	Maximum	Mean = Minimum	Mean = Maximum
*Micronutrients*						
Phosphorus	12.68	(9.83, 15.53)	55	151	*t* = −30.04, *p* = 0.000	*t* = −98.17, *p* = 0.000
Magnesium	6.27	(4.75, 7.80)	15	45	*t* = −11.56, *p* = 0.000	*t* = −51.29, *p* = 0.000
Iron	4.64	(3.98, 5.30)	1.8	2.7	*t* = 8.73, *p* = 0.000	*t* = 5.96, *p* = 0.000
Calcium	9.39	(6.42, 12.36)	55	151	*t* = −31.08, *p* = 0.000	*t* = −96.51, *p* = 0.000
*Macronutrients*						
Lipids	13.39	(10.09, 16.69)	5	7	*t* = 5.14, *p* = 0.000	*t* = 3.91, *p* = 0.000
Protein	13.85	(11.93, 15.76)	2.5	3.0	*t* = 11.99, *p* = 0.000	*t* = 11.46, *p* = 0.000
Energy	354.56	(329.21, 379.90)	520	550	*t* = −13.20, *p* = 0.000	*t* = −15.60, *p* = 0.000

*Note:* For each nutrient, the first *t* test (Min) assesses whether the observed mean differs significantly from the minimum Codex Alimentarius standard for RUTF, and the second *t* test (Max) assesses whether the observed mean differs significantly from the maximum standard. Statistical significance was assessed at *p* < 0.05. This table shows the mean values and confidence intervals for micronutrient and macronutrient contents in locally produced ready‐to‐use therapeutic foods (RUTFs) compared with the Codex Alimentarius standards.

## 4 Discussion

This study aimed to compare ingredients and nutritional values of locally formulated food preparations used in the four provinces most affected by COVID‐19 with those of the standard formulations recommended for treating SAM. We found uniformly low compliance among 40 locally formulated food preparations: none met ≥ 50% of nutrient targets. While macronutrient levels were generally adequate or above standard, micronutrient deficiencies predominated.

While the formulations assessed in this study have not yet aligned with WHO/Codex Alimentarius requirements, their ingredient profiles closely mirror those reported in other studies. The only two formulations exceeding one quarter of WHO requirements used soy and maize flours as their base. Owino et al. (2014) developed milk‐free and peanut‐free local alternative RUTF, soybean–maize–sorghum (SMS) RUTF prototypes as an alternative to standard RUTF in Kenya, using linear programming to optimize palatability and nutrient adequacy. The alternative was found to be palatable, well tolerated, and culturally acceptable to school‐aged children. Shelf stability was maintained for at least 12 months under ambient storage (Owino et al. 2014).

Some non‐standard RUTF can still improve nutrition outcomes. Similarly, Irena et al. (2015) in Zambia, developed a milk‐free and peanut‐free, soybean‐maize‐sorghum RUTF. Following the prototype, SMS RUTF was produced by Valid Nutrition for testing. The alternative was not inferior to the standard on recovery rates, weight gain, and mortality (Irena et al. 2015). Bahwere et al. (2016) in DRC showed that a peanut‐free, fish‐enriched RUTF is non‐inferior to the standard on recovery, weight gain, and treatment success among SAM children. These three examples demonstrate that locally available, peanut‐free, milk‐free, or fish‐enriched options can be great alternatives for SAM management in low‐resource settings. These examples align with UNICEF Supply Division's typology of “renovation” (legume/cereal substitutions for peanuts that still meet WHO protein‐source guidance), “innovation” (introduction of fish, egg, or insect proteins to reduce or replace milk), and “novel” (full removal of milk proteins with amino‐acid fortification as needed) (UNICEF and WHO 2021). It is important to note that our study also included milk‐free and peanut‐free recipes.

Emerging evidence demonstrates that alternative RUTFs can result in significant cost reductions compared to traditional peanut and milk‐based versions, with powdered milk alone comprising nearly half the production expense (Danso and Tewfik 2025). Low‐dairy or dairy‐free formulas, such as soy‐maize‐sorghum (SMS) RUTF, can be produced at much lower costs around US$1583 per metric ton versus US$2393 for the standard formula (Akinmoladun et al. 2023). Savings are also evident when substituting locally sourced legumes, cereals, or animal proteins (Pajak et al. 2025). In Ethiopia, for instance, RUTFs made from chickpea or millet lowered ingredient expenses by 35%–50%, and fish‐based blends in Colombia reduced costs by about 20%, all while sustaining acceptance and recovery outcomes (Akinmoladun et al. 2023). Sourcing ingredients locally further trims transportation and import fees, leading to reported savings of 14%–52% and boosting regional supply chains (Pajak et al. 2025). Strategies like reducing RUTF dosage can add an additional 15%–20% in savings per child (Danso and Tewfik 2025). Collectively, cereal‐legume, milk‐free, and low‐dairy RUTFs yield cost benefits of 14% to over 50% without sacrificing therapeutic effectiveness. Long‐term studies, however, are still needed to confirm ongoing recovery and micronutrient sufficiency (Pajak et al. 2025).

From a macronutrient perspective, our locally produced formulas generally met or exceeded WHO recommendations for protein (2.5–3 g/100 kcal) and fat (5–7 g/100 kcal), aligning with experiences across Africa and Asia. However, energy density was sometimes modest, and fat content occasionally exceeded the usual range. While excess protein is metabolized and excreted, surplus fats are stored in the body. Although a higher fat intake can support rapid catch‐up growth, sustained exposure may raise concerns about long‐term cardiometabolic health in children recovering from SAM, highlighting the importance of optimizing fat quality and post‐recovery monitoring (Rand et al. 2003; FAO/WHO 2022; Akomo et al. 2019; Bahwere et al. 2017; Das et al. 2018). The principal response at a public health level involves two strategies: first, bolster regional food technology and apply linear programming to create community‐acceptable products from local ingredients (e.g., soy, maize, amaranth) that consistently meet protein and energy criteria; second, harmonize procurement and quality standards with Codex guidelines, strengthen local production, and maintain emergency reserves to safeguard supply against disruptions like border closures or pandemics that have repeatedly limited dairy access in the DRC (UNICEF and WHO 2021; FAO/WHO 2022; Bahwere et al. 2016; Fetriyuna et al. 2023). Practical actions include setting fat content ceilings, informing caregivers about dietary fat quality for children, and implementing monitoring of SAM survivors for early signs of non‐communicable diseases. Theoretical considerations highlight a trade‐off between fast recovery and future health risks, while policy implications stress the need for collaboration between the IMAM and prevention of chronic illnesses.

Far more severe in our findings were gaps in micronutrients: key vitamins both water‐soluble (especially B12 and folate) and fat‐soluble (A, D, E, K) as well as crucial minerals (calcium, zinc, iodine, iron, selenium), frequently fell below recommended targets. This trend, also reported in recent reviews of alternative RUTFs using cereal‐legume bases and vegetable oils, is concerning (Pajak et al. 2025; Danso and Tewfik 2025; Akinmoladun et al. 2023). B12 is largely found in animal sources and predictably becomes scarce when milk powder is reduced or omitted; while folate occurs in legumes, processing often diminishes its levels, meaning milk‐free options often do not reach WHO/Codex standards, raising the risk of impaired blood formation, neurodevelopment, and cognition if not properly fortified (Danso and Tewfik 2025; Akinmoladun et al. 2023; Pajak et al. 2025). Removing dairy fats also strips away natural sources of vitamins A and D, and using vegetable oils does not guarantee adequate levels of A, D, E, or K unless they are specifically added at stable, active concentrations and shielded from oxidation, potentially leaving children prone to infections, rickets, blood‐clotting issues, and vision problems despite suitable weight gain (Pajak et al. 2025; Akinmoladun et al. 2023). Plant‐based ingredients offer less mineral density and, due to phytic acid, reduced absorbability of minerals like zinc, iron, and calcium; even where hemoglobin levels rise as seen in some SMS studies ensuring overall mineral sufficiency is inconsistent without targeted fortification and processing to minimize anti‐nutrients (via dehulling, fermenting, or sprouting) (Bahwere et al. 2017; Danso and Tewfik 2025; Akinmoladun et al. 2023).

To maintain both the cost and acceptance benefits of local alternatives, programs must mandate fortification with crystalline B12 and folic acid; preformed vitamins A, D3, E, and K1; and bioavailable mineral salts (zinc gluconate, potassium iodate, calcium carbonate). These should be paired with (FAO/WHO 2022; WHO 2021; Pajak et al. 2025; Danso and Tewfik 2025) regular product quality control at the central level (factory) and at users' end level (at home, sample taken from child's plate) (Mekonnen et al. 2018). Some formulations (e.g., fish–based or soy–based) have been linked to improvements in hemoglobin and iron status, especially in anemic children (Danso and Tewfik 2025). While iron status has improved in some trials, vitamin A and zinc repletion have not consistently matched that of standard RUTFs, highlighting the need for careful fortification strategies (Akinmoladun et al. 2023).

In practice, these measures bridge critical nutritional gaps while retaining local ingredient sourcing; theoretically, they illustrate that therapeutic equivalence hinges on bioavailability and micronutrient metabolism as much as on macronutrient sufficiency. From a policy perspective, this calls for mandatory fortification, regional premix supply chains, financial incentives for local producers, and tighter alignment with Codex to ensure that innovative products are available at scale and quality (UNICEF and WHO 2021; FAO/WHO 2022). These strategies address persistent “hidden hunger,” reinforce the need for holistic nutritional recovery, and urge DRC policymakers to establish regulatory frameworks and regional supply networks for standard fortification.

Effective dissemination of these findings is crucial. They should be shared with nurses in charge of OTNUs through targeted workshops and accessible summaries, enabling them to adapt counseling, treatment, and referral practices. For instance, OTNU staff should be trained to spot at‐risk children—those who may still face vitamin or mineral deficiencies despite weight gain—and tailor feeding guidance accordingly. Provincial and national decision‐makers will receive policy briefs outlining the economic and health risks posed by insufficiently fortified local alternatives and showing feasible solutions for fortification and ingredient optimisation. This two‐tiered communication is essential in fragile settings like the DRC, where both frontline practitioners must update practices promptly and policymakers must align national regulations and resources with Codex Alimentarius standards.

The key strength of this study is its mixed‐methods nutrient assessment: we combined (i) theoretical profiling using NutVal with (ii) laboratory analyses for selected micronutrients. By sampling real products used in OTNUs during the COVID‐19 period, we generated context‐specific evidence on non‐industrial, locally formulated food preparations evidence which has been largely absent in the DRC and identified opportunities and operational challenges for milk‐free, peanut‐free locally formulated food preparation in resource‐limited settings.

This work has some limitations. First, the sample size was modest and drawn from four provinces, representing RUTFs produced within that specific geographic area. Second, due to budget constraints, we did not quantify all 22 priority nutrients and the majority of micronutrients were assessed only theoretically. Future studies should pair full laboratory panels with NutVal for all nutrients to enable complete theory‐vs‐assay comparisons. Third, NutVal modeling depends on daily ration inputs; in our setting, these quantities were based on nurse‐prescribed rations rather than precisely measured intakes.

## 5 Conclusion

This study provides essential insights into the disparities between locally formulated food preparations and the standards recommended by WHO. The food preparations assessed do not yet match (unbalanced macro and micronutrient content) WHO/Codex requirements and need important upgrading before being used for children with SAM. They underscore the potential for enhancing therapeutic strategies in the management of SAM. It is crucial to engage local industrial actors engaged in food processing and researchers in local production initiatives to ensure that these formulations adhere to international nutritional standards while incorporating locally sourced and cost‐effective ingredients. Future enhancements should prioritize the correction of micronutrient deficiencies, the balance of macronutrient contents, and the optimization of energy density in accordance with international guidelines. Furthermore, scaling locally formulated preparations should proceed only with mandatory fortification standards aligned to Codex/WHO, reliable regional premix supply chains, and strengthened regulatory oversight of quality and fat composition. Frontline OTNU staff need practical guidance, briefs and training to detect and manage residual micronutrient risks and to tailor counseling accordingly. At the system level, investing in local ingredient value chains (soy, maize, fish, amaranth) can reduce import dependency and bolster resilience during crises, while prospective cohorts and full nutrient panels should monitor long‐term outcomes (including cardiometabolic and neurodevelopmental health). This, in turn, would strengthen the efforts of initiatives aimed at addressing unresolved cases of severe acute malnutrition in resource‐limited settings.

## Author Contributions

**Marc Bosonkie:** conceptualization (lead), data curation (lead), formal analysis (lead), investigation (lead), methodology (lead), writing – original draft (lead). **Celestin Nzanzu Mudogo:** methodology (supporting), writing – review and editing (supporting). **Hannah Silverstein:** supervision (supporting), writing – review and editing (supporting). **Olufunmilayo I. Fawole:** validation (supporting), writing – review and editing (supporting). **Gael Compta:** formal analysis (supporting), methodology (supporting), writing – review and editing (supporting). **Koto‐Te‐Nyiwa Ngbolua:** formal analysis (supporting), methodology (supporting), writing – review and editing (supporting). **Tesky Koba:** formal analysis (supporting), writing – review and editing (supporting). **Ruphin Mbuyi:** validation (supporting), writing – review and editing (supporting). **Berthold Bondo:** writing – review and editing (supporting). **Paul‐Samsom Lusamba:** conceptualization (supporting), supervision (supporting), writing – review and editing (supporting). **Mala Ali Mapatano:** conceptualization (lead), methodology (supporting), supervision (supporting), validation (supporting), writing – review and editing (supporting).

## Ethics Statement

The authors have nothing to report.

## Consent

All procedures performed in studies involving human participants were in accordance with the ethical standards of the national research committee (Kinshasa School of Public Health IRB) and with the 1964 Helsinki Declaration. Informed consent was obtained from all individual participants included in the study.

## Conflicts of Interest

The authors declare no conflicts of interest.

## Supporting information


**Table S1:** Macro‐ and micronutrient profiles of the 40 analyzed samples (laboratory results).

**Table S2:** Macro‐ and micronutrient profiles of the 40 recipes estimated using NutVal.

## Data Availability

The data used for analysis can be accessed upon reasonable request by writing an email to the corresponding author.
